# Label-Free Microdroplet Concentration Detector Based on a Quadruple Resonant Ring Metamaterial

**DOI:** 10.3390/s26031013

**Published:** 2026-02-04

**Authors:** Wenjin Guo, Yinuo Cheng, Jian Li

**Affiliations:** 1 School of Information and Communication Engineering, North University of China, Taiyuan 030051, China; 2 Department of Electronic Engineering, Taiyuan Institute of Technology, Taiyuan 030024, China; 3 School of Information and Communication Engineering, University of Electronic Science and Technology of China, Chengdu 610054, China

**Keywords:** metamaterials, label-free, trace, detector

## Abstract

This paper proposes and experimentally validates a label-free microdroplet concentration detector based on a quad-resonator metamaterial. The device exploits the linear relationship between the dielectric constant of a binary mixed solution and its concentration, mapping concentration information to absorption frequency shifts with a sensitivity of 28.53 GHz/RIU. System modeling was performed through full-wave simulation. Experimental results demonstrate a highly linear relationship between resonance frequency shift and concentration across ethanol, water, and ethanol–water solutions. The relative deviation between simulation and measurement is less than 3%, validating the model’s reliability and the robustness of the detection principle. This detector supports rapid non-contact sample replacement without requiring chemical labeling or specialized packaging. It can be mass-produced on standard PDMS substrates, with each unit reusable for >50 cycles. With a single measurement time of <30 s, it meets high-throughput detection demands. Featuring low power consumption, high precision, and scalability, this device holds broad application prospects in point-of-care diagnostics, online process monitoring, and resource-constrained scenarios. Future work will focus on achieving simultaneous multi-component detection via multi-resonator arrays and integrating chip-level wireless readout modules to further enhance portability and system integration.

## 1. Introduction

Metamaterials are artificially engineered subwavelength-structured materials [1] whose electromagnetic properties far exceed those of conventional natural materials. These properties are primarily influenced by factors such as topological structure and unit cell dimensions [2]. In recent years, electromagnetic metamaterials have found extensive applications in both military and civilian domains, particularly in electromagnetic stealth [3], perfect lenses [4], antennas [5], and wave splitting [6,7]. Due to their high sensitivity to environmental changes through electromagnetic resonance, metamaterials also hold numerous applications in medical [8], chemical [9,10], and biological detection [11,12] fields. For instance, in liquid concentration detection, distinct dielectric constants of liquids with varying concentrations within the same microstructure can generate unique resonance frequencies [13], enabling highly sensitive concentration sensing. Researchers placed the test liquid in the gap between the copper plate of the metamaterial absorber and the rear cavity resonator to detect methanol and ethanol solutions of varying concentrations in the Ku-band. Furthermore, by placing the test container directly on the detector substrate, liquid concentration can be perceived based on the resonance curve. By comparing liquid detectors based on complementary open-loop resonators (CSRR) [14], extended-gap SRRs (EG-SRR) [15], and circular SRRs (Circular-SRR) [16], this paper proposes a sample-embedded structure that enables label-free, trace, and rapid concentration detection of methanol and ethanol–water solutions.

This study designed a quad-resonant circular-square ring liquid concentration detector to detect liquids at varying concentrations. To achieve this, dielectric constant models were first established for methanol–water, ethanol–water, and methanol–ethanol solutions at different concentrations. During measurement, the test liquid was injected into the liquid test tube on the detector surface for simulation analysis, with simulation results validated through actual testing. The results demonstrate excellent agreement between simulation and experimental data. The detector’s liquid loading method eliminates the need for chemical reagent detection with the sample, facilitates sample replacement, and enables multiple measurements within a short timeframe. Furthermore, the proposed detector offers advantages such as simple operation, reusability, compact structure, low cost, and ease of fabrication, showcasing promising application prospects.

## 2. Principles and Models

**Figure 1:**
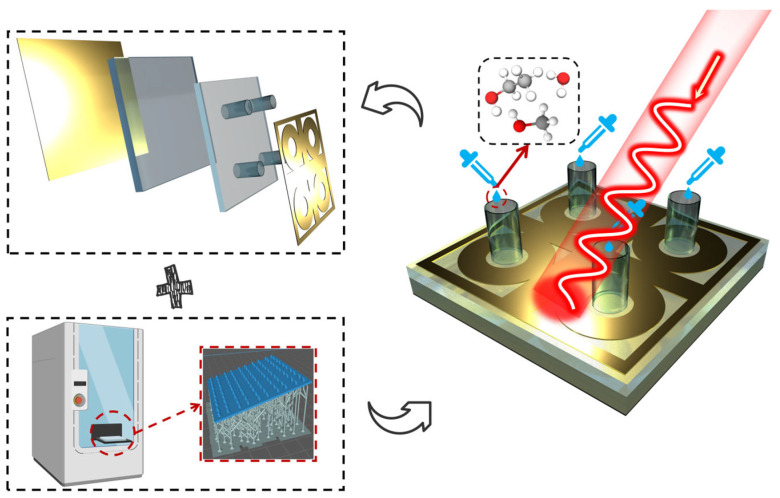
Schematic diagram of liquid detector effect.

**Figure 2:**
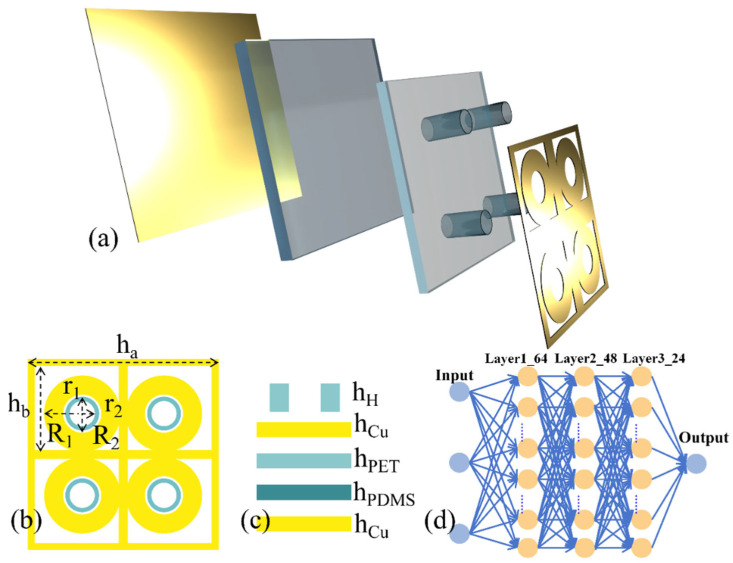
(**a**) Schematic Diagram of Unit Structure Layering, (**b**) Unit Parameter Diagram, (**c**) Schematic Diagram of Interlayer Thickness, (**d**) Schematic Diagram of Deep Learning Network.

This paper designs a four-ring cross-shaped liquid concentration detector. Its schematic diagram is shown in Figure 1, its structure is illustrated in Figure 2, and its specific parameters are listed in Table 1. The dielectric substrate is 1.8 mm thick PDMS with a dielectric constant ε = 2.75 and loss tangent tanθ = 0.003. All copper layers have a thickness of 0.035 mm and a conductivity of 5.8 × 10^7^ S/m [6]. The tested solutions comprised methanol, ethanol, and ethanol–water mixtures at varying concentrations. To analyze the impact of different liquid concentrations on detector resonance, each tested liquid was divided into four concentration groups spanning 20% to 80% concentration with 20% increments. The dielectric constants for different concentrations, as provided in Reference [13], conform to the Debye model [17]. Modeling and simulation were performed using the microwave simulation software CST 2023. A waveguide port was employed for feeding, with X and Y boundary conditions set as unit cell and Z boundary conditions set as Open.

Inspired by traditional neural networks [18], deep learning models [19], and genetic algorithms [20], this paper proposes an efficient optimization framework based on deep neural networks (DNNs) to replace the time-consuming iterative process in gigahertz metamaterials (GHz MMs) design within conventional commercial 3D full-wave simulation software. The constructed DNN comprises three fully connected layers, with the first and second layers containing 64 and 48 neurons, respectively, both subject to L2 regularization. This regularization strategy effectively suppresses overfitting, significantly reducing the training dataset size while maintaining generalization performance on the validation set. The network adopts an encoder–decoder architecture: the fully connected layers perform a single up-sampling step, transforming the 26-length structural parameter vector into a 170-length latent space feature. Subsequently, three layers of transposed convolutions complete the remaining up-sampling task, enhancing the correlation between adjacent frequency points through learnable correlation filters. The final convolutional layer further smooths the predicted spectrum. Sixteen sampling points are clipped from each end of the output tensor to eliminate edge effects caused by zero-padding, yielding an absorption spectrum with 320 frequency points. The dataset comprises 27,328 “structural parameter-absorption spectrum” samples generated via CST full-wave simulation. The dataset is split into training (19,130), validation (5465), and test (2733) sets at a 7:2:1 ratio. The model was implemented in a Python/PyCharm 2021 environment using the Adam optimizer for end-to-end training, with single-GPU training taking 987.53 s. On the test set, the Mean Absolute Error (MAE) was 0.004 and the Root Mean Square Error (RMSE) was 0.005, validating the dual advantages of accuracy and efficiency in the proposed method.

(1) $$Ps=\left[\begin{array}{c} \begin{array}{ccc} h_{a} & h_{b} & \begin{array}{ccc} R_{1} & R_{2} & \begin{array}{ccc} r_{1} & r_{2} & \begin{array}{ccc} h_{H} & h_{Cu} & \begin{array}{cc} h_{PET} & h_{PDMS} \end{array} \end{array} \end{array} \end{array} \end{array}\\ \begin{array}{c} \begin{array}{ccc} h_{a}^{1} & h_{b}^{1} & \begin{array}{ccc} R_{1}^{1} & R_{2}^{1} & \begin{array}{ccc} r_{1}^{1} & r_{2}^{1} & \begin{array}{ccc} h_{H}^{1} & h_{Cu}^{1} & \begin{array}{cc} h_{PET}^{1} & h_{PDMS}^{1} \end{array} \end{array} \end{array} \end{array} \end{array}\\ \begin{array}{c} \ldots \ldots \\ \begin{array}{c} \begin{array}{ccc} h_{a}^{n-1} & h_{b}^{n-1} & \begin{array}{ccc} R_{1}^{n-1} & R_{2}^{n-1} & \begin{array}{ccc} r_{1}^{n-1} & r_{2}^{n-1} & \begin{array}{ccc} h_{H}^{n-1} & h_{Cu}^{n-1} & \begin{array}{cc} h_{PET}^{n-1} & h_{PDMS}^{n-1} \end{array} \end{array} \end{array} \end{array} \end{array}\\ \begin{array}{ccc} h_{a}^{n} & h_{b}^{n} & \begin{array}{ccc} R_{1}^{n} & R_{2}^{n} & \begin{array}{ccc} r_{1}^{n} & r_{2}^{n} & \begin{array}{ccc} h_{H}^{n} & h_{Cu}^{n} & \begin{array}{cc} h_{PET}^{n} & h_{PDMS}^{n} \end{array} \end{array} \end{array} \end{array} \end{array} \end{array} \end{array} \end{array} \end{array}\right],$$

**Figure 3:**
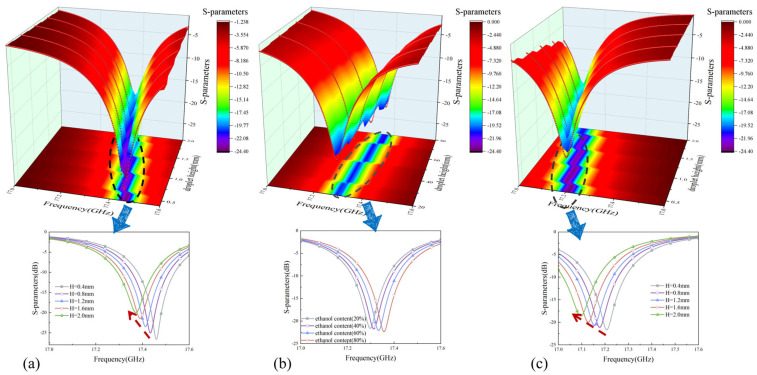
Liquid step detection diagrams. (**a**) Ethanol height step result diagram, (**b**) ethanol percentage step diagram, and (**c**) water drop height step percentage diagram.

To further validate the predictive reliability of the deep neural network (DNN) model, three sets of outstanding structural parameters were manually selected from all prediction results and listed in Table 2. All three candidate schemes simultaneously met the dual optimization objectives of absorption rate and frequency shift, preliminarily confirming the effectiveness of the DNN model. Among them, Predicted_1 exhibits the smallest prediction error (|δ| < 2%), while Predicted_2 and Predicted_3 show relative errors of 4.1% and 4.7%, respectively, slightly higher than the former. After comprehensively evaluating absorption intensity, frequency shift magnitude, and prediction accuracy, Predicted_1 was ultimately selected as the structure for experimental validation. Its spectral response characteristics are shown in Figure 3: Resonance peaks appear within the target frequency band, with peak absorption rate and corresponding frequency shift both reaching optimal values. This aligns well with simulation results, further validating the accuracy of the DNN model and the optimality of Predicted_1.

The proposed metamaterial liquid detector is rigorously formulated within the framework of transmission-line theory [21]. The perfectly reflecting back plane of the detector is equivalent to a short-circuited stub, so that the input impedance Zin is obtained by the parallel combination of the metasurface impedance and the substrate impedance. The complex effective surface impedance Zeff of the metasurface layer is expressed as [22]:

(2) $$Z_{eff}=Re\left(Z_{eff}\right)+Im\left(Z_{eff}\right)=M+jX,$$

In Equation (2), M and X denote the real and imaginary parts of the effective surface impedance, respectively, and j is the imaginary unit. The relationship between the input impedance Zin and the real and imaginary parts of the metasurface’s effective impedance, M and X, is expressed as [23]:

(3) $$Z_{in}=\frac{j(M-X)Z_{0}sin(\beta d)}{\sqrt{\varepsilon_{r}}Mcos\left(\beta d\right)+j\left[\sqrt{\varepsilon_{r}}Mcos\left(\beta d\right)+Z_{0}sin(\beta d)\right]},$$

In Equation (3): Z_0_ = 377 Ω is the wave impedance in free space; ε_r_ is the relative permittivity of the dielectric layer; $\beta =2\pi f\sqrt{\varepsilon_{r}}/c$ is the phase constant of the incident electromagnetic wave in the dielectric, where f is the frequency of the incident wave and c is the speed of light in vacuum; d is the thickness of the dielectric layer.

Subsequently, we can obtain the following formula [24]:

(4) $$A=1-{\left|S_{11}\right|}^{2}-{\left|S_{21}\right|}^{2}\approx 1-{\left|S_{11}\right|}^{2},$$

(5) $$S_{11}=\frac{-\sqrt{\varepsilon_{r}}\left[Mcos\left(\beta d\right)+Xsin\left(\beta d\right)\right]+j[\left(M-Z_{0}\right)\sin\left(\beta d\right)-\sqrt{\varepsilon_{r}}Xcos(\beta d)]}{\sqrt{\varepsilon_{r}}\left[Mcos\left(\beta d\right)-Xsin\left(\beta d\right)\right]+j[\left(M+Z_{0}\right)\sin\left(\beta d\right)-\sqrt{\varepsilon_{r}}Xcos(\beta d)]},$$

This paper proposes and validates a microwave-band micro-droplet sensor synergistically composed of periodic metamaterial resonators and engineered dielectric substrates, exhibiting outstanding electromagnetic absorption properties. Through deep learning system optimization of resonator geometric parameters, the device demonstrates exceptionally high responsivity to both the volume and type of minute liquids, enabling precise quantitative analysis of ethanol volume fraction under label-free conditions. The resonance frequency shift in absorption peaks forms a robust label-free conversion mechanism. This strategy extends beyond ethanol detection, laying a technological foundation for constructing multifunctional sensing platforms applicable to diverse scenarios, including food safety monitoring [25,26], environmental pollution tracing [27,28], and point-of-care biomedical diagnostics [29,30,31]. Further improvements in sensitivity and parallel detection capabilities through topological optimization and arrayed designs are anticipated, accelerating the commercial deployment of label-free microfluidic detection technology.

## 3. Analysis and Discussion of Results

To ensure the reliability of experimental data, strict control measures were implemented for sample preparation, introduction, and stability during testing. For the standard solutions (methanol, ethanol, and ethanol–water binary mixtures), concentration accuracy was guaranteed through multi-step operations: (1) Concentration gradients (20%, 40%, 60%, 80% by volume) were designed based on the linear response characteristics of liquid dielectric constant to concentration (consistent with the Debye model [13]), ensuring concentration changes could stably induce resonant frequency shifts. (2) Solutions were prepared using precision pipettes (accuracy ±0.1 μL) via volume ratio matching. For binary mixtures, a fixed total volume was maintained, with solvents and solutes added proportionally. After preparation, solutions were allowed to stand for 30 min to ensure uniform mixing, avoiding local concentration deviations. (3) Dielectric constant parameters of solutions with different concentrations were referenced from experimentally validated standard data [13], consistent with input values in the CST simulation model. The relative deviation between simulation and experimental results (<3%) indirectly verified the accuracy of solution preparation.

The introduction of solutions into the microchannel adopted the detector’s sample-embedded structure. Specifically, the detector surface was integrated with a dedicated liquid test tube, precisely aligned with the resonant ring structure to ensure effective coupling between the liquid and metamaterial surface. A micro-syringe was used to inject 10–50 μL of standard solution directly into the test tube, with special attention to avoiding bubble generation (bubbles would cause uneven dielectric distribution and interfere with resonant signals). This method enabled non-contact rapid sample replacement without special packaging or pretreatment, with a single injection operation taking <10 s, meeting high-throughput detection requirements.

Regarding the potential evaporation risk of volatile components (ethanol) during measurement, targeted control strategies were adopted: (1) The single detection cycle (<30 s) is much shorter than the time scale for significant ethanol evaporation (complete evaporation of 50 μL ethanol at room temperature requires several minutes), with an evaporation amount of <1% during measurement—insufficient to cause detectable concentration changes. (2) The micro-volume sample (10–50 μL) and semi-closed test tube structure reduced the solution-air contact area, further lowering the evaporation rate. (3) All experiments were conducted in a constant temperature and humidity laboratory (25 ± 1 °C, 50 ± 5%RH) to avoid accelerated evaporation due to temperature fluctuations. (4) The measurement process was optimized: detection was initiated immediately after solution injection, and samples were quickly removed post-test to avoid prolonged standing. Meanwhile, each concentration sample was measured in parallel three times, with the average value taken as the final result to offset random errors from potential micro-evaporation. Experimental verification showed that the resonant frequency shift fluctuation of the same standard solution in three consecutive measurements (total time < 3 min) was <0.1 GHz, confirming that evaporation’s impact on detection accuracy was effectively controlled.

Figure 3 shows the absorption peak response patterns of the metamaterial liquid detector simulated by CST under pure water, ethanol, and water–ethanol mixtures. As shown in Figure 3a,c, the main absorption peak exhibits a monotonic red shift with increasing liquid column height in the microfluidic channel, and the displacement is positively correlated with the liquid column height. This result quantitatively verifies the device’s high sensitivity in trace liquid detection scenarios. Figure 3b further demonstrates that when the ethanol volume fraction in the solution increases from 20% to 80%, the absorption peak position undergoes a significant blue shift, confirming the detector’s precise identification capability for binary liquid components.

**Figure 4:**
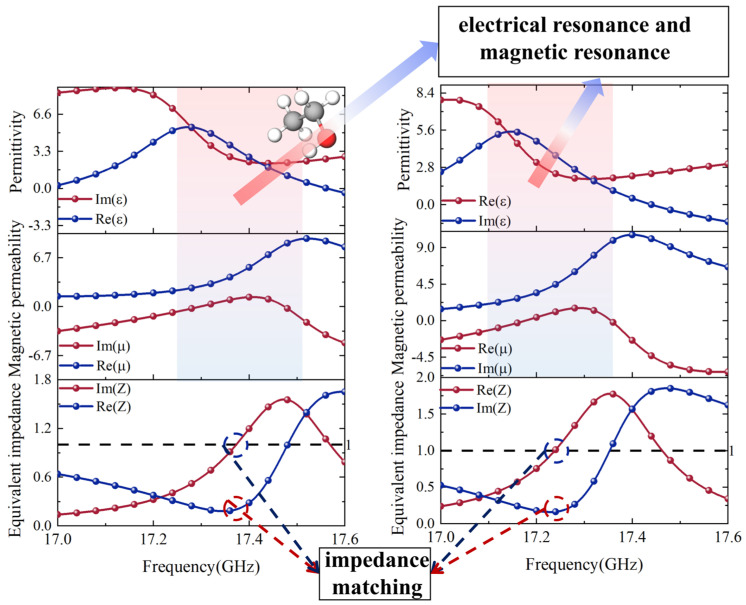
Equivalent parameter diagrams for detectors with water and ethanol additions.

**Figure 5:**
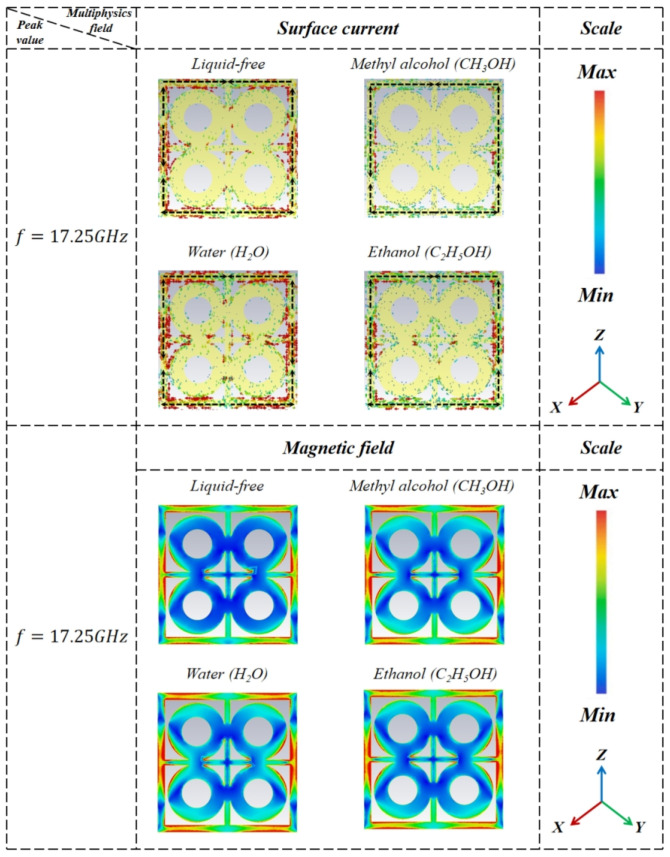
Magnetic field and surface current diagram.

To further elucidate the working mechanism of the metamaterial liquid detector, this paper conducts a systematic analysis from three dimensions: equivalent electromagnetic parameters, magnetic field distribution, and surface current modes. The results are shown in Figure 4 and Figure 5. Comparing the two figures reveals that in the high-absorption region, strong electric dipole and magnetic dipole resonances coexist [32]. This manifests as electric field energy concentrating in the resonator gap, while magnetic field energy is primarily localized at the edges of the resonant ring. As shown in Equation (4), when the real part of the equivalent impedance is 1 and the imaginary part is 0, the system achieves impedance matching [3], reflections approach zero, and the absorption coefficient reaches its peak. This result is in perfect agreement with simulation and experimental findings.

Subsequent analysis of the surface magnetic field and surface current revealed that the peripheral surface current is divided into three segments on both the left and right sides. Under conditions without liquid and in the presence of methanol, the current distribution patterns are similar. Specifically, the currents in the upper and lower segments flow along the z-axis from negative to positive, while the current in the middle segment flows in the exact opposite direction to those in the upper and lower segments. Additionally, the current directions on the upper and lower sides are also opposite. On the upper side, currents converge toward the central axis from both sides, whereas on the lower side, currents diverge from the central axis toward both sides. However, in the absence of liquid, the magnitude of the surface current was greater than that detected in the presence of methanol.

**Figure 6:**
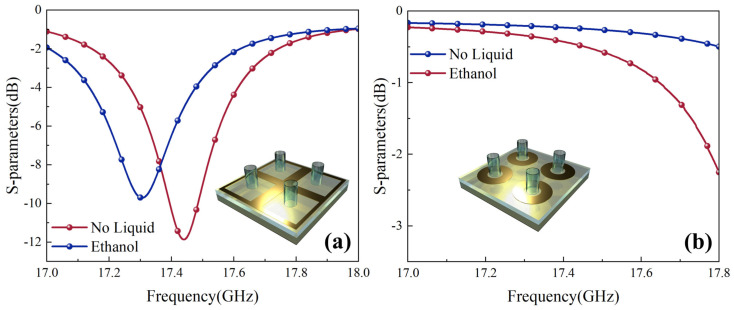
(**a**) Detector results after losing the resonant ring; (**b**) detector results after losing the outer square ring.

Furthermore, this paper systematically investigates the coupling effect of the surface copper ring. As shown in Figure 6, removing the resonant circular ring not only causes a significant blue shift in the absorption peak but, more critically, leads to a rapid decline in the detector’s sensitivity toward the target liquid. If the outer square ring is further removed, the device completely loses its detection capability. These results indicate that the circular resonant ring primarily enhances detection sensitivity by strengthening localized field confinement, while the outer square ring maintains electromagnetic coupling and resonant integrity, ensuring the overall detection functionality is realized.

**Figure 7:**
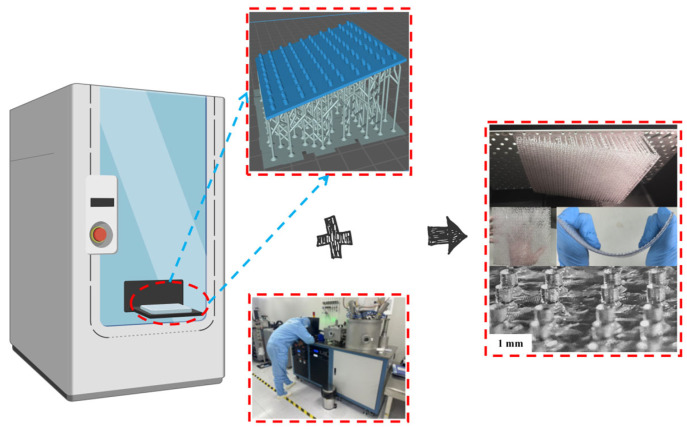
Process and physical diagram.

**Figure 8:**
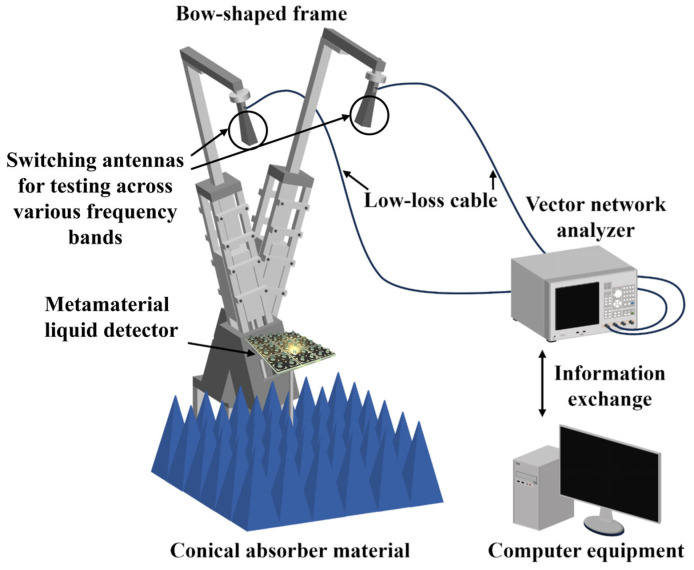
Test schematic diagram.

Meanwhile, we also conducted preparation and testing in the laboratory. The specific process for copper sputtering onto PET film is as follows: First, prepare 1 mm thick PET film and magnetron sputtering equipment, including a vacuum chamber, copper target, power supply, vacuum pump, and gas supply system. The PET film undergoes cleaning treatment using a cleaning solution: a NaOH solution and surfactant to remove surface impurities and minor oxides. Subsequently, the PET surface is optimized through charge adjustment and other methods to enhance adhesion for the subsequent copper layer. Next, the PET film is mounted on the base of the magnetron sputtering equipment. The vacuum chamber is sealed, and the vacuum pump is activated to evacuate the chamber to a vacuum level of approximately 0.5 Pa. Argon gas is then introduced as the working gas, with pressure adjusted to an appropriate range (typically 0.1–1 Pa). To control the copper layer thickness to 0.035 mm, high-energy argon ions bombard the copper target under a strong electric field, causing copper atoms to sputter and deposit onto the PET film surface. After sputtering, remove the PET copper film from the equipment and rinse it with water to remove surface residues. To enhance the corrosion resistance of the copper layer, a passivation solution can be used to passivate the copper surface. Finally, place the treated PET copper film in an oven and dry it at 105 °C. Next, the PDMS base solution and curing agent are mixed at a 10:1 mass ratio and stirred thoroughly to eliminate bubbles. The mixed PDMS solution is then spin-coated evenly onto the non-copper side of the PET copper film. The spin-coating speed and duration are controlled via the spin-coating equipment to ensure uniformity of the PDMS layer. Subsequently, the spin-coated PET copper film is placed in an oven for curing at the set temperature until the PDMS layer fully solidifies, achieving a cured thickness of approximately 1.8 mm. Finally, a 0.035 mm thick copper foil is sputter-coated onto the non-copper side of the fabricated sample using the same process described above. Finally, as shown in Figure 7. Ultimately, comparison with simulation results revealed a high degree of consistency between physical and simulated outcomes, with errors remaining within acceptable limits. The test schematic is shown in Figure 8. At the same time, we compared relevant work as shown in Table 3.

## 4. Conclusions

This paper proposes, fabricates, and validates a label-free microdroplet concentration detector based on a quad-resonator metamaterial. Building upon a model where the dielectric constant of a binary mixed solution varies with concentration, this model is embedded into a full-wave simulation. Experiments and simulations simultaneously monitored resonant frequency shifts. Across the entire concentration range, simulations closely matched experimental results (relative deviation < 3%), validating the model’s reliability and the robustness of the detection principle. Additionally, this detector enables rapid, non-contact sample replacement without any chemical reagents, capable of performing dozens of measurements within minutes. The device is reusable and requires no special encapsulation; it can be fabricated in bulk on standard PDMS substrates. This resonator offers significant advantages for low-power, precise concentration detection in point-of-care diagnostics, online process monitoring, and resource-constrained scenarios. Future work will focus on multi-resonator arrays for simultaneous multi-component detection and integration of on-chip wireless readout to further enhance portability.

## Figures and Tables

**Table 1:** Structural parameters table.

Structure Parameter (mm)				
h_a_	h_b_	R_1_	R_2_	r_1_
9.6	4.1	2	1	0.8
r_2_	h_H_	h_Cu_	h_PET_	h_PDMS_
0.5	2	0.035	1	1.8

**Table 2:** Parameter Set.

Model	Number	Structure Parameter									
		h_a_	h_b_	R_1_	R_2_	r_1_	r_2_	h_H_	h_Cu_	h_PET_	h_PDMS_
DNN	Predicted_1	9.6	4.1	2	1	0.8	0.5	2	0.035	1	1.8
	Predicted_2	9.6	4.1	2.1	1.1	0.9	0.5	2	0.035	1	1.8
	Predicted_3	9.6	4.1	1.9	0.9	0.8	0.5	2	0.035	1	1.8

**Table 3:** Performance comparison chart.

References	Types	Core Metrics
[33]	SPR	1115 RIU^−1^
[34]	SPR	34.62 RIU^−1^
[35]	metamaterial	1.08 THz/RIU
This work	metamaterial	28.53 GHz/RIU

## Data Availability

All data that support the findings of this study are included within the article.
